# Multilevel analysis to identify the factors associated with caesarean section in Bangladesh: evidence from a nationally representative survey

**DOI:** 10.1093/inthealth/ihac006

**Published:** 2022-02-22

**Authors:** Md Sabbir Ahmed, Mansura Islam, Ishrat Jahan, Imran Faisal Shaon

**Affiliations:** Department of Community Health and Hygiene, Faculty of Nutrition and Food Science, Patuakhali Science and Technology University, Dumki, Patuakhali-8602, Bangladesh; Department of Sociology, Faculty of Social Science, University of Dhaka, Dhaka 1000, Bangladesh; Department of Food Microbiology, Faculty of Nutrition and Food Science, Patuakhali Science and Technology University, Dumki, Patuakhali-8602, Bangladesh; Department of Animal Nutrition, Faculty of Animal Husbandry, Bangladesh Agricultural University, Mymensingh 2202, Bangladesh

**Keywords:** caesarean section, maternal health, MICS-2019, multilevel modelling

## Abstract

**Background:**

Caesarean delivery has a significant role in reducing maternal and child death. However, unnecessary utilization has adverse health effects. This study aimed to assess the prevalence and associated factors of caesarean delivery in Bangladesh.

**Methods:**

Data from the latest Bangladesh Multiple Indicator Cluster Survey (MICS, 2019) was used in this study. Since MICS data are hierarchical in nature, multilevel modelling was used.

**Results:**

The prevalence of caesarean section (CS) was 67.4% among Bangladeshi women. Multilevel analysis suggests the age of the women, household wealth status, utilization of antenatal care (ANC) , delivery at a health facility and division were significantly associated with CS. Women who delivered in a private health facility had the highest odds for CS (odds ratio [OR] 10.35 [95% confidence interval {CI} 8.55 to 12.54]). Women 30–34 y of age had a 36% higher likelihood of CS compared with women 15–19 y of age (OR 1.36 [95% CI 1.03 to 1.79]). The odds of CS positively increased with household wealth status. Women who had at least one ANC visit had a 1.7 times higher possibility of CS (OR 1.70 [95% CI 1.26 to 2.30]).

**Conclusions:**

Policy guidelines on caesarean deliveries are urgently needed in Bangladesh to avoid unnecessary caesarean deliveries and protect mothers from the consequences.

## Introduction

Caesarean section (CS) is a surgical procedure that is usually performed when vaginal delivery is not possible or may put the baby and/or mother at risk.^1,2^ According to the World Health Organization (WHO), the rate of CS delivery should be 10–15% on a population level.^3^ Worldwide about 19% of deliveries are done by CS and the rates are increasing.^4^ According to a previous study based on the Bangladesh Demographic and Health Survey (BDHS) 2014, about 23.9% of Bangladeshi children were delivered by CS.^1^

Proper utilization of CS can play a vital role in reducing maternal and child deaths during delivery. However, several adverse effects on maternal and child health and well-being have been reported due to unnecessary CS deliveries.^5,6^ During 2018, The Lancet published a series on optimising caesarean section use and highlighted the short-term and long-term effects of CS on the health of women and children.^7^ According to The Lancet, adverse impacts of CS on maternal health include a risk of uterine rupture, abnormal placentation, ectopic pregnancy and stillbirth. In addition, it is evident that babies delivered by CS have different hormonal, physical, bacterial and medical exposures that may adversely affect neonatal physiology.^7^ Previously it was reported that women mainly prefer CS due to a lack of knowledge on CS delivery, misinformation on vaginal delivery, fear, anxiety and labour pain.^1,8^ Alarmingly, a large portion of Bangladeshi women undergo CS delivery and decided for CS about 1 month before their expected delivery date.^1,9^ According to Save the Children, during 2016–2018, the rate of CS increased by 51% in Bangladesh, most of which were unnecessary. They also reported that in 2018 about US$483 million dollars in out-of-pocket expenses were paid for medically unnecessary CSs.^10^ This poses an economic burden not only for the family, but also for the national economy.^11^

Previous efforts have been made to identify the trends and factors affecting CS delivery in Bangladesh.^1,2,9^ The prevalence of CS increased almost eightfold in Bangladesh during 2000–2014.^9^ The most recent country-representative data on the utilization of CS delivery came from the latest Bangladesh Multiple Indicator Cluster Survey (MICS; 2019).^12^ It is therefore important to fill the research gap and report the latest statistics to assist in initiating immediate public health actions. Further identification of risk factors following an advanced statistical analysis approach is crucial for policymakers and public health activists to understand where progress has been made, where to invest additional resources and to design evidence-based intervention packages. Therefore this study aimed to assess the prevalence and associated factors of caesarean delivery in Bangladesh according to most recent national representative data.

## Methods

### Data

This study was based on a secondary data source. Data were extracted from the Bangladesh MICS 2019, which was a country-representative cross-sectional survey. Since the mid-1990s, MICS has become the largest source of statistically sound and internationally comparable data on children and women worldwide. Trained field workers conducted face-to-face interviews with household members on a variety of topics related to maternal and child health.

The Bangladesh MICS 2019 was part of the six-round global MICS conducted by the Bangladesh Bureau of Statistics (BBS) and supported by the United Nations Children's Fund (UNICEF). Survey data were collected from 64 administrative districts in Bangladesh at the household (HH) level. To ensure national representation, a two-stage stratified random sampling procedure was followed to collect data from the households. The urban–rural areas within each district were considered as the main sampling strata. Within each stratum, a specific number of census enumeration areas (EAs) were systematically selected with a probability proportional to size. After listing HHs within selected EAs, a systematic sample of 20 HHs was drawn from each primary sampling unit (PSU). Details of the sampling process, data collection procedure and questionnaire are available in the final survey report.^12^

### Study variables

Delivery by CS was considered as the outcome variable for this study. The data were collected by asking the women, ‘Was (name of the last child) delivered by caesarean section?’ The responses were recorded as yes=1 or no=0.

The demographic and socio-economic status (SES) of the women and their respective households were considered as independent variables for this study. As we intended to perform a two-level multilevel analysis, all the independent variables included in this study are of two types: level 1 and level 2. Level 1 or individual-level variables include age: women's age in years in 5-y intervals from 15 to 49 y; educational status: highest educational level or grade attended; household wealth status: computed by principal component analysis based on household assets and materials used to build the house; received antenatal care (ANC) at least once during the last pregnancy; delivery in a private health facility: yes or no; watch television (almost every day): yes or no; and pregnancy was wanted: yes or no. Level 2 or community-level variables include place of residence: urban or rural; and division: the administrative region of Bangladesh.

### Statistical analysis

The data set was cleaned (i.e. removing missing cases, coding, recoding variables etc.) before formal data analysis. Descriptive statistics were run to calculate the number and frequency. Pearson's χ^2^ test was performed to compare the prevalence of CS across different independent variables. Since MICS data are hierarchical in nature, that is, individuals (women) are nested within households and households are nested within a higher level (clusters), multilevel modelling (MLM) was appropriate compared with a traditional binary logistic regression model.^13^ For our multilevel multivariable regression analysis, we fitted four models: model 1 (null model) was fitted without explanatory variables to assess the variance in the outcome of interest between communities, model 2 was fitted for individual-level variables, model 3 was fitted for community-level variables and model 4 (final model) was fitted for both individual- and community-level variables. The measure of community variation was estimated as the intraclass correlation coefficient (ICC) and an ICC value >0 was considered adequate to conduct the MLM.^14^ For measures of variation we also calculated the median odds ratio (MOR) and proportional change in variance (PCV).^15^ Variables showing a p-value <0.25 in the bivariate analysis were selected for multivariable analysis.^16^ The effect of predictive variables was measured using odds ratios (ORs) with 95% confidence intervals (CIs). Akaike's information criterion (AIC) was used to assess the goodness of fit of each model. Multicollinearity between independent variables was checked using the variance inflation factor (VIF). None of the variables showed multicollinearity problems (VIF <10). All statistical tests were two-sided and considered significant at p<0.05. Data were analysed using Stata version 14.2 (StataCorp, College Station, TX, USA).

## Results

Complete data for 4903 women (weighted) were included in this study. The majority of the participants were between 20 and 24 y of age (34.2%), completed secondary education (53.8%), lived in rural areas (72.7%), delivered in a private health facility (70.2%) and watch television almost every day (64.4%) (Table 1).

**Table 1.** Demographic and SES of the study participants and the prevalence of caesarean delivery.

		Caesarean delivery			
Variables	Participants, n (%)	Yes, n (%)	No, n (%)	χ^2^	p-Value
Individual-level characteristics					
Age (years)					
15‒19	676 (13.8)	436 (64.5)	240 (35.5)	22.89	0.006*
20‒24	1677 (34.2)	1105 (65.9)	573 (34.1)		
25‒29	1362 (27.8)	953 (70.0)	409 (30.0)		
30‒34	837 (17.1)	592 (70.7)	245 (29.3)		
35‒39	288 (5.9)	177 (61.5)	111 (38.5)		
40‒49	63 (1.3)	43 (68.3)	20 (31.7)		
Educational status					
Pre-primary or none	203 (4.1)	113 (55.7)	90 (44.3)	108.11	<0.001*
Primary	762 (15.6)	440 (57.7)	323 (42.3)		
Secondary	2636 (53.8)	1742 (66.1)	894 (33.9)		
Higher secondary or higher	1301 (26.5)	1010 (77.6)	291 (22.4)		
Household wealth status					
Poorest	508 (10.4)	257 (50.6)	251 (49.4)	103.82	<0.001*
Poor	714 (14.6)	450 (62.9)	265 (37.1)		
Middle	940 (19.2)	637 (67.8)	303 (32.2)		
Rich	1191 (24.3)	809 (67.9)	382 (32.1)		
Richest	1549 (31.6)	1152 (74.3)	398 (25.7)		
Received ANC (at least once)					
No	304 (6.2)	154 (50.7)	150 (49.3)	41.13	<0.001*
Yes	4598 (93.8)	3151 (68.5)	1448 (31.5)		
Delivery in private health facility					
No	1462 (29.8)	514 (35.2)	948 (64.8)	969.13	<0.001*
Yes	3440 (70.2)	2791 (81.1)	650 (18.9)		
Watch television (almost every day)					
No	1736 (35.4)	1034 (59.6)	702 (40.4)	74.13	<0.001*
Yes	3167 (64.6)	2271 (71.7)	896 (28.3)		
Pregnancy was wanted					
Yes	3844 (78.4)	2612 (68.0)	1232 (32.0)	2.35	0.187
No	1058 (21.6)	693 (65.4)	366 (34.6)		
Community-level characteristics					
Residence					
Rural	3540 (72.7)	2352 (66.4)	1189 (33.6)	5.46	0.065
Urban	1362 (27.8)	953 (70.0)	409 (30.0)		
Division					
Barishal	190 (3.9)	131 (69.3)	58 (30.7)	155.59	<0.001*
Chattogram	1026 (20.9)	583 (56.8)	443 (43.2)		
Dhaka	1376 (28.1)	1040 (75.6)	336 (24.4)		
Khulna	661 (13.5)	487 (73.8)	173 (26.2)		
Mymensingh	238 (4.9)	156 (65.5)	82 (34.5)		
Rajshahi	612 (12.5)	435 (71.1)	177 (18.9)		
Rangpur	493 (10.1)	321 (65.1)	172 (34.9)		
Sylhet	307 (6.3)	151 (49.2)	156 (50.8)		
Overall	4903 (100)	3305 (67.4)	1598 (32.6)		

*Significant at p<0.05.

### Prevalence of caesarean delivery

**Figure 1.**
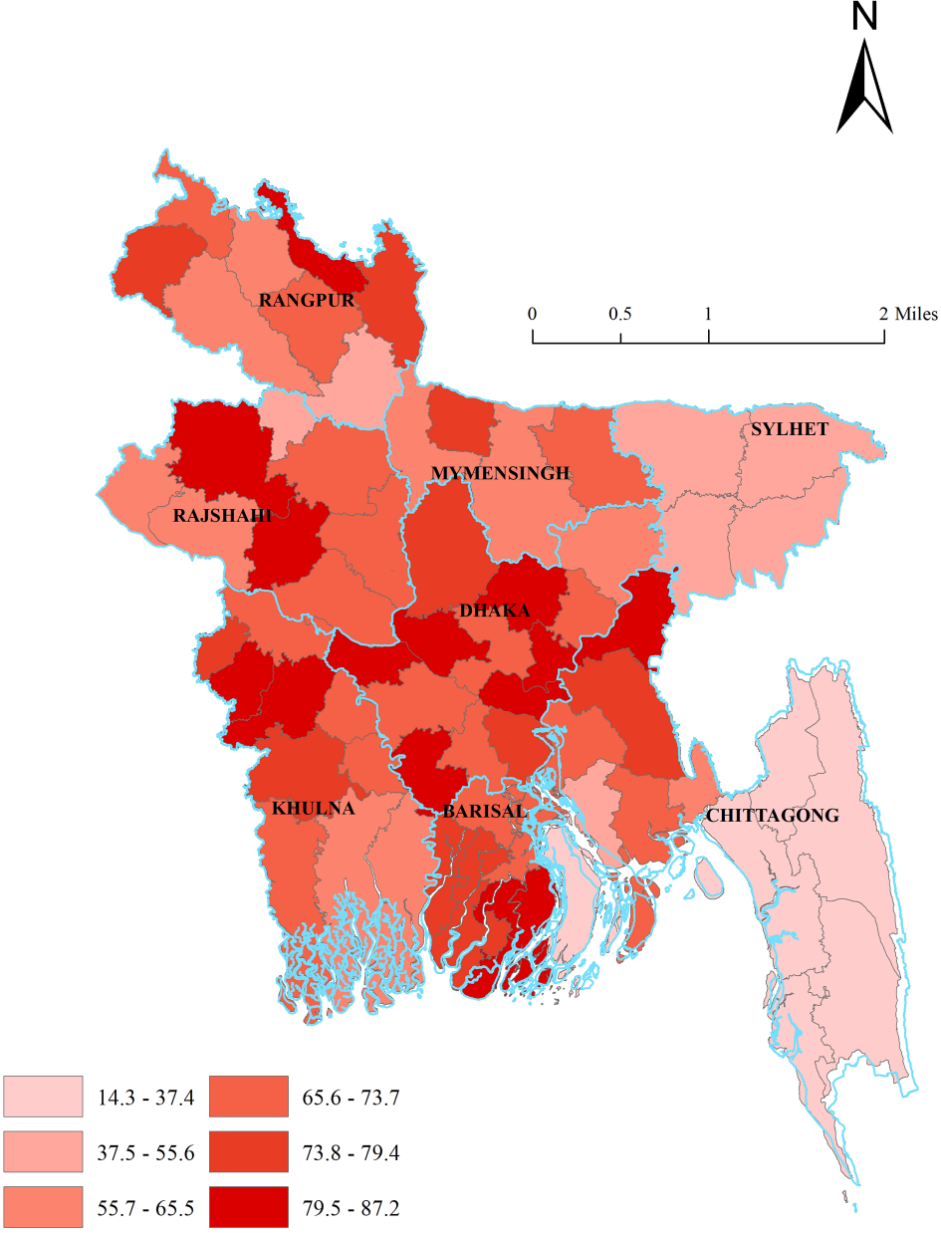
District level map of CS in Bangladesh.

As shown in Table 1, the prevalence of utilizing CS was 67.4% (n=3305) in Bangladesh. The prevalence was higher among women 30–34 y of age (70.7%), completed higher secondary or higher educational grades (77.6%), living in urban areas (70.0%), Dhaka division (75.6%), richest households (74.3%), received at least one ANC visit (68.5%), gave birth at a private health facility (81.1%), watch television almost every day (71.7%) and pregnancy was wanted (68.0%) (Table 1). More than half of the women (57.7%) reported that their CS was decided before labour pains began (data not shown). As depicted in Figure 1, overall, southern and western Bangladesh have the higher rates of CS, and among the 64 administrative districts in Bangladesh, Jhinaidah (87.2%) had the highest and Bandarban (14.3%) had the lowest rate of CS (Supplement 1).

### Factors associated with caesarean delivery

As shown in Table 2, the null model (model 1) reveals that clustering exists in determining CS. The ICC value of the null model indicates approximately 21.5% of the variance in the outcome is a result of factors contributing at the community level. The final model (model 4) revealed significant variances and the MOR of 2.1 showed the effect of community heterogeneity (suggesting that if a women moved to a community with a higher probability of CS, the median increase in the odds of CS would be 2.1-fold). Additionally, 32.3% of the variance is the odds of CS across communities explained by both individual- and community-level factors, as indicated by the PCV.

**Table 2.** Factors associated with CS in Bangladesh.

Variables	Model 1, OR (95% CI)	Model 2, OR (95% CI)	Model 3, OR (95% CI)	Model 4, OR (95% CI)
Individual-level characteristics				
Age (years)				
15‒19		Ref.		Ref.
20‒24		1.04 (0.81 to 1.33)		1.08 (0.85 to 1.38)
25‒29		1.16 (0.90 to 1.50)		1.22 (0.95 to 1.56)
30‒34		1.37 (1.03 to 1.82)*		1.36 (1.03 to 1.79)*
35‒39		1.00 (0.69 to 1.46)		1.05 (0.72 to 1.51)
40‒49		1.32 (0.62 to 2.77)		1.22 (0.59 to 2.51)
Educational status				
Pre-primary or none		Ref.		Ref.
Primary		0.89 (0.58 to 1.37)		0.86 (0.56 to 1.31)
Secondary		1.13 (0.75-1.69)		1.08 (0.72 to 1.61)
Higher secondary or higher		1.69 (1.10 to 2.61)*		1.51 (0.98 to 2.33)
Household wealth status				
Poorest		Ref.		Ref.
Poor		1.28 (0.96 to 1.72)		1.26 (0.95 to 1.67)
Middle		1.21 (0.90 to 1.62)		1.32 (0.99 to 1.76)
Rich		1.19 (0.88 to 1.60)		1.34 (1.00 to 1.80)*
Richest		1.24 (0.91 to 1.70)		1.54 (1.11 to 2.15)**
Received ANC (at least once)				
No		Ref.		Ref.
Yes		1.75 (1.29 to 2.39)***		1.70 (1.26 to 2.30)***
Delivery in private health facility				
No		Ref.		Ref.
Yes		11.64 (9.57 to 14.16)***		10.35 (8.55 to 12.54)***
Watch television (almost every day)				
No		Ref.		Ref.
Yes		1.30 (1.08 to 1.55)		1.18 (0.99 to 1.41)
Pregnancy was wanted				
Yes		Ref.		Ref.
No		0.99 (0.82 to 1.20)		0.96 (0.79 to 1.15)
Community-level characteristics				
Residence				
Rural			Ref.	Ref.
Urban			1.15 (0.96 to 1.37)	0.99 (0.81 to 1.22)
Division				
Barishal			Ref.	Ref.
Chattogram			0.47 (0.33 to 0.65)***	0.42 (0.29 to 0.60)***
Dhaka			1.36 (0.98 to 1.90)	1.18 (0.82 to 1.69)
Khulna			1.15 (0.82 to 1.61)	0.99 (0.69 to 1.43)
Mymensingh			0.77 (0.48 to 1.24)	1.15 (0.69 to 1.92)
Rajshahi			0.90 (0.63 to 1.28)	0.93 (0.63 to 1.36)
Rangpur			0.89 (0.57 to 1.71)	0.99 (0.67 to 1.46)
Sylhet			0.33 (0.22 to 0.49)***	0.48 (0.31 to 0.75)**
Measure of variation				
Variance (SE)	0.902 (0.149)	0.688 (0.159)	0.610 (0.129)	0.430 (0.142)
ICC (%)	21.53	17.31	15.65	11.57
PCV (%)	Ref.	23.72	32.37	52.32
MOR	2.46	2.19	2.10	1.86
Model fit statistics				
AIC	5989.08	4871.60	5866.87	4798.72

Ref.: reference category; SE: standard error.

^a^Model 1 (null model) was fitted without determinant variables.

^b^Model 2 is adjusted for individual-level variables only.

^c^Model 3 is adjusted for community-level variables only.

^d^Model 4 is adjusted for both individual- and community-level variables.

*p<0.05, **p<0.01, ***p<0.001.

Multilevel analysis suggests the age of the woman, household wealth status, utilization of ANC, delivery at a health facility and division were significantly associated with CS among Bangladeshi women. Women who delivered in a private health facility had the highest odds for CS (OR 10.35 [95% CI 8.55 to 12.54]). Women 30–34 y of age had 36% higher likelihood of CS compared with women 15–19 y of age (OR 1.36 [95% CI 1.03 to 1.79]). The odds of CS positively increased with household wealth status. Women who received at least one ANC visit had a 1.7 times higher possibility for CS (OR 1.70 [95% CI 1.26 to 2.30]). Women residing in the Chattogram and Sylhet divisions had 58% and 52% less likelihood of CS, respectively, compared with the Barishal division (OR 0.42 [95% CI 0.29 to 0.60] and 0.48 [0.31 to 0.75], respectively).

## Discussion

In this study we reported the latest prevalence of CS in Bangladesh and its associated demographic and socio-economic factors. The prevalence of CS in Bangladesh increased >2.5-fold compared with earlier national statistics^1^ and >6-fold compared with the WHO recommendation.^3^ According to our findings, two in every three Bangladeshi women and their babies are at risk of adverse effects of CS.^5,6^ The prevalence of CS in Bangladesh was found to be highest among the South and Southeast Asian women.^17^ In the public health facilities of India, Pakistan and Afghanistan, the prevalence of CS has been reported as 13.7%,^18^ 13.1%^19^ and 10.2%,^20^ respectively. The rate was 37.9%,^18^ 25%^19^ and >15%^21^ in the private health facilities of India, Pakistan and Nepal, respectively. According to the 2014 BDHS report, 54.8% of CSs were decided before the delivery date.^1^ We found that during 2019, about 57.7% of CSs were decided before labour pains began, which clearly indicates that the number of intentional CSs is increasing in Bangladesh. Several medical and non-medical factors are reported to be associated with the increasing use of CS. Among the medical factors, maternal age, obesity, multiple gestation, diabetes, high blood pressure, hypertension/pre-eclampsia, delivery-related complications and lack of ANC are identified as being responsible for higher proportions of CS births.^18,22^ On the other hand, non-medical factors that are factors for the increase in CSs are place of residence, education, improved economic status, the large number of private hospitals and unethical acts of the doctors in these hospitals, preference of patients and changes in cultural and social factors and demand for CSs.^18,23,24^

Regarding the risk factors, women ages 30–34 y had a 36% higher possibility of CS compared with women ages 15–19 y. Older women are at higher risk for caesarean delivery because they are more likely to have comorbidities.^25^ The literature has also shown that regardless of whether labour is spontaneous or induced, older women are more likely to undergo caesarean delivery because of higher rates of induction, particularly elective induction.^26^ We also noted that a majority of adolescent mothers (15–19 y) go through CS, which is very alarming. It is well studied that the rate of early marriage as well as early pregnancy is very high in Bangladesh. This finding indicates that adolescent mothers are not physically strong or mature enough to deliver a baby and choose CS. However, as we do not have enough data to support this statement, further studies are warranted to explain the scenario.

The likelihood of CS positively increased with the household wealth status of the women. Higher wealth status may influence CS for safe childbirth, as they have the economic stability to bear the expenses related to surgery. Another aspect is that women from higher socio-economic backgrounds are more likely to use private facilities, which opens up the possibility of higher CS rates in these facilities. That also explains how private maternal care facilities are making a profit from unnecessary CSs.^27^ Although we do not have enough data to explain this, future mixed-methods studies may provide necessary information. The participants who are currently living in the Chattogram and Sylhet divisions had lower odds for CSs compared with women from the Barishal division. The reasons for these divisional disparities in utilizing CS are not clear. One possible explanation could be women from these regions are more aware of the consequences of unnecessary CSs^5^ and the benefits of vaginal delivery.^28^

The women who received at least one ANC visit during their pregnancy had lower odds for a CS; a similar association is also reported by a systematic review.^29^ As recommended by the WHO, ANC visits are crucial to identify complications in advance.^30^ Uncertainty about the child's situation in the pregnancy makes mothers apprehensive, leading many to choose CS delivery.^31^ During ANC visits, discussion of the risks and benefits of both normal delivery and CS helps women make informed decisions.^32^

Delivery in a private health facility had the greatest influence on CS in Bangladesh. Private health facilities are mainly profit-driven and want to protect themselves from unexpected delivery risks.^33^ Other factors that influence the rate of CS at private facilities are a perception of improved healthcare, availability of qualified obstetricians or gynaecologists, available drugs and diagnosis facilities.^9^ Ensuring universal health coverage for all and increasing adequate facilities in government health facilities may improve the scenario. However, further studies are warranted to explore the influence of private facility delivery on CS in Bangladesh.

This study is not without limitations. A major limitation of this study is the cross-sectional nature of the data, as temporal relationships cannot be explained with such data. Another limitation is the inability to incorporate all potential independent variables.^1,2,9^ Despite limitations, the study used the most recent nationwide data, so study findings can be generalized at the national level.

## Conclusions

The prevalence of CS was found to be very high in Bangladesh. The age of the women, educational status, administrative division, household wealth status, ANC (at least one) and delivery in a private health facility were independent predictors of CS among Bangladeshi women.

## Data Availability

Data set used in this study is freely available at https://mics.unicef.org/surveys.
